# Rates of hematophagous ectoparasite consumption during grooming by an endemic Madagascar fruit bat

**DOI:** 10.1186/s13071-018-2918-1

**Published:** 2018-06-01

**Authors:** Riana V. Ramanantsalama, Aristide Andrianarimisa, Achille P. Raselimanana, Steven M. Goodman

**Affiliations:** 1 Mention Zoologie et Biodiversité Animale, Université d’Antananarivo, BP 906, (101) Antananarivo, Madagascar; 2 Association Vahatra, BP 3972, (101) Antananarivo, Madagascar; 3 Field Museum of Natural History, 1400 South Lake Shore Drive, Chicago, Illinois 60605 USA

**Keywords:** Diptera, Nycteribiidae, Pteropodidae, *Rousettus madagascariensis*, Madagascar, Host-parasite interactions

## Abstract

**Background:**

Few details are available on the consumption of ectoparasites, specifically bat flies (Diptera: Nycteribiidae and Streblidae), by their chiropteran hosts while grooming. Such details are important to document consumption rates of ectoparasites by their bat host provide details on the dynamics of host-parasite interactions. We present data on ectoparasite consumption rates for an endemic Malagasy fruit bat (Pteropodidae: *Rousettus madagascariensis*) occupying a cave day roost colony in northern Madagascar. Using quantified behavioral analyses, grooming and associated ingestion rates were measured from infrared videos taken in close proximity to day-roosting bats. The recorded individual bats could be visually identified to age (adult, juvenile) and sex (male, female), allowing analyses of the proportion of time these different classes allocated to consuming ectoparasites *via* auto-grooming (self) or allo-grooming (intraspecific) per 10 min video recording session. These figures could then be extrapolated to estimates of individual daily consumption rates.

**Results:**

Based on video recordings, adults spent significantly more time auto-grooming and allo-grooming than juveniles. The latter group was not observed consuming ectoparasites. Grooming rates and the average number of ectoparasites consumed per day did not differ between adult males and females. The mean extrapolated number consumed on a daily basis for individual adults was 37 ectoparasites. When these figures are overlaid on the estimated number of adult *Rousettus* occurring at the roost site during the dry season, the projected daily consumption rate was 57,905 ectoparasites.

**Conclusions:**

The details presented here represent the first quantified data on bat consumption rates of their ectoparasites, specifically dipterans. These results provide new insights in host-parasite predation dynamics. More research is needed to explore the mechanism zoonotic diseases isolated from bat flies might be transmitted to their bat hosts, specifically those pathogens that can be communicated *via* an oral route.

**Electronic supplementary material:**

The online version of this article (10.1186/s13071-018-2918-1) contains supplementary material, which is available to authorized users.

## Background

The past decade has seen an important increase in studies of bats as reservoirs of diverse diseases [1–3]. Another facet of this research has shown that bat ectoparasites, specifically hematophagous bat flies of the families Streblidae and Nycteribiidae, are possible reservoirs and vectors for different zoonotic diseases, e.g. bacteria such as *Bartonella* and *Rickettsia* [3–5]; viruses such as rhabdovirus [6, 7]; and protozoans including the hematoparasite *Polychromophilus* [8, 9]. Hence, there is indirect evidence that bat ectoparasites may be responsible for the transmission of infectious diseases to their hosts, although it remains to be demonstrated that this could be *via* an oral route, specifically direct consumption of ectoparasites by bats.

Although there are growing literature data on the importance of ectoparasites as prey and the associated epidemiological implications [10, 11], only a few studies that have documented ingestion rates by bats of their ectoparasites. A project conducted on New World streblids found evidence that the fruit bat *Artibeus jamaicensis* (Phyllostomidae) may actively feed on its bat flies of the genus *Megistopoda* [12]. Further, captive *A*. *jamaicensis* readily accepted and consumed *Megistopoda* bat flies and the remains of streblids were identified in the stomachs of different species of Costa Rican bats [12]. Further, in Malaysia, the bat *Megaderma spasma* (Megadermatidae) feeds actively on *Eoctenes spasmae* (Cimicidae) ectoparasites [13]. Hence, there is indeed evidence that bats feed on their dipteran parasites, but quantitative details on occurrence and frequency are lacking in the literature.

The purpose of this paper is to present quantified behavioral observations on the proportion of time while in the day roost site an endemic Malagasy fruit bat grooms and actively consumes ectoparasites overlaid on possible age and sex differences. Extrapolations are made as to the average daily ectoparasite consumption rates per individual bat. These data provide new insights into host-ectoparasite predation dynamics, as well as a possible means zoonotic diseases isolated from bat flies, specifically those communicated through oral means, might be transmitted to their hosts through the consumption of ectoparasites.

## Methods

### Study area

Data were collected between 29 June to 10 July 2017 at a day roost site of *Rousettus madagascariensis* (Chiroptera: Pteropodidae) in the Grotte des Chauves-souris (12°57.4'S, 49°7.1'E), Parc National d’Ankarana in the far north of Madagascar. Ankarana is characterized by limestone karst formations and the natural habitat is dry deciduous forest [14]. The study was conducted during the dry season, and estimates are available for this period on the number and age ratio of *R*. *madagascariensis* occupying the day roost site in the same cave [15, 16].

### Video recording

Data on grooming behavior, specifically bat feeding rates on ectoparasites, were calculated *via* the analysis of recordings made with an infrared video camcorder [1080p HD Infrared Night Vision and Full Spectrum Camcorder (Cleveland Paranormal Supply Co., Mentor, OH, USA)] employing an infrared light for supplementary illumination [Evolva Future Technology T38 IR 38 mm lens infrared flashlight light night vision torch (Shenzhen Dikesai International Trade Co., Ltd, Guangdong)]. The peak intensity wavelengths of these apparatus are 810 nm. The camcorder, which was connected to a remote control, was placed at a fixed place within the cave, approximately 4 m from the target *Rousettus madagascariensis* group, with the intent in following individual bat activities. Tourists visit the cave, which disrupts the natural behavior of this species [17], and the video recordings were made in an area of the cave outside the typical visitor circuit.

Each day of behavioral recordings made in the cave, sessions commenced at 6:15 h, 7:15 h, 8:15 h, 13:15 h, 14:15 h, and 15:15 h. Recordings were made for 10 min per session, which on a daily basis resulted in one hour of recording (six sessions × 10 min). The sessions commencing at 6:15 h and 13:15 h, when the recording material was installed during a period of about 30 min, were followed by 15 min of no activity near the roosting bats, to allow them to calm down after being disturbed. The start and termination of the video recording for the other sessions were controlled with the remote device.

The methods for data sampling and processing followed a previous behavioral study on *R*. *madagascariensis* in the same cave [17]. Video recordings were reviewed on a computer screen measuring 40.5 × 16.2 cm, and scored based on behavioral variables using a scan sampling method [18, 19]. An ethogram (Table 1), derived from previous studies of non-Malagasy bat species [20, 21], was created to categorize and quantify the behavior of *R*. *madagascariensis*.

**Table 1:** Ethogram of behavior for the scan sampling methods

Behavior categories	Description
Rest	Not moving any part of the body
Crawl	Alternate placement of feet or/and thumb claws to shift position on the rock face
Groom	Scratching or licking the body or/and wing phalanges
Fight	Biting or hooking (rapid swiping with thumb claw) the opponent
Consume ectoparasites	Taking ectoparasites into the mouth, masticating, and no evidence of rejecting them

### Scan sampling

A scan sampling technique was used to document different behavioral states of target individuals within a group and to calculate an individual's time activity budget (i.e. the proportion of time spent in an activity) [17]. One scan sampling consisted of following five individual bats based on the recorded video, each independently for three seconds and for 10 rounds of observation. Given that in certain cases, an individual disappeared from or others flew into view, two other groups of five individuals were also followed during the 10 min period. The proportion of time each individual performed each of the following behavioral activities was calculated: rest, crawling into or outside of the focal cluster, inter-bat aggression (“fighting”), auto-grooming (self-grooming), allo-grooming (associated with the body of a neighboring animal), and consuming ectoparasites on its own body *via* auto-grooming (Additional file 1: Video S1) or a neighboring animal *via* allo-grooming (Additional file 2: Video S2).


**Additional file 1**: **Video S1.** Consumption of ectoparasites by adult male *Rousettus madagascariensis via* auto-grooming. (WMV 10725 kb)



**Additional file 2**: **Video S2.** Consumption of ectoparasites by adult male *Rousettus madagascariensis via* allo-grooming. (AVI 7806 kb)


On the basis of recorded video, the sequence of steps that bats seized ectoparasites and consumed them is as follows: detainment with their teeth, tongue, or lips; mastication; and then swallowing individual bat flies (Additional file 1: Video S1, Additional file 2: Video S2). Based on analysis of 10 hours of recordings, we have no evidence of bats spitting out invertebrates or consume more than one individual per feeding bout.

### *Rousettus* ectoparasites

Based on 3577 bat flies collected from 639 individual *R. madagascariensis* in the same cave, two species were identified [22, 23]: one streblid (*Megastrebla wenzeli*) and one nycteribiid (*Eucampsipoda madagascariensis*). The streblid, capable of flight, represented 9.8% of the bat flies identified, and the wingless nycteribiid, the remaining 90.2%. Both of these ectoparasites are host-specific to *R*. *madagascariensis* with a prevalence of 97.1%, a mean intensity of 8.7 bat flies per host individual, and a mean abundance of 5.5 and 7.9 bat flies for adult female and male of *R. madagascariensis*, respectively [22, 24]*.* Apart from these two dipteran families, the only other ectoparasites found on *R. madagascariensis* were minute Acari, but no quantified information is available on levels of parasitism [25].

### Determination of *Rousettus* age and sex classes

In the context of the recordings made of roosting bats, two age classes were established, largely based on body size: smaller juveniles and distinctly larger adults. The sex of an individual was determined based on its general body form and associated external sexual organs, with males having a visible scrotum-like structure, with either abdominal or descended testicles, and females with evidence of an indented pelvis and in some cases enlarged mammae. In 14 cases, lactating females with neonates attached to their breast were within the framed area of video recording. However, as these neonates were hidden under the wings of their mothers, no behavior information was recorded for either individual.

### Data analysis

For each 10 min scan sample, the mean proportion of time the different age classes (adult and juvenile) and sex classes (male and female) spent conducting the five types of behavior (Table 1) were calculated. The Spearman’s rank correlation for data with non-normal distributions was employed using SPSS version 21 [26], to examine the correlation between grooming and ectoparasites consumption, stratified by age and sex classes*.* The Wilcoxon rank sum test was performed using R 3.4 [27], to examine possible differences in grooming behavior and ectoparasites consumption between age and sex classes. The Kruskall-Wallis test with *post-hoc* Dunn tests and the Wilcoxon rank test with R 3.4 [27], were both employed to investigate the relation between session periods and the consumption of ectoparasites. Based on these calculations, it was possible to estimate the number of ectoparasites consumed by individual bats per day. In turn, these data were superimposed on an estimate of the number of *Rousettus* occupying the cave, taking into account the proportion of adults in relation to the juveniles during the same seasonal period as this study [15, 16], to estimate the number of ectoparasites consumed per day within the colony.

## Results

### Time activity budget for each behavior category

In total, 68 sessions of 10 min were performed, giving rise to approximately 11 hours of video recordings. Only 20 min of recording were carried out on 29 June 2017 and 60 min each day thereafter until 10 July 2017. As the data were collected during the dry season, about five to six months after the birthing season, the proportion of juveniles was high, but less than adults (Table 2). The bat sex ratio of filmed and analyzed individuals was biased in favor of males represented by the two age classes. The number of females with neonates excluded from the dataset (*n* = 14 individuals) was insufficient to account for the skewed sex-ratio.

**Table 2:** The proportion of time spent by *Rousettus madagascariensis* associated with each type of behavior. Mean ± standard deviation (minimum-maximum values) of the proportion of time spent by *R. madagascariensis*, separated into different age and sex classes, associated with each type of behavior. These values are based on six recording sessions per day, each 10 min, for a daily total of 60 min, and over nine days

Age and sex classes	No. of observations	Rest (%)	Crawl (%)	Fight (%)	Auto-grooming (%)	Allo-grooming (%)	Consuming ectoparasites *via* auto-grooming (%)	Consuming ectoparasites *via* allo-grooming (%)
Juvenile	48	93.6 ± 17.5 (0–100)	2.3 ± 2.7 (0–10)	0	3.3 ± 10.4 (0–1.4)	0.8 ± 2.0 (0–8.2)	0	0
Adult	84	73.4 ± 14.3 (20–100)	0.7 ± 1.1 (0–5)	3.2 ± 2.6 (0–30)	14.2 ± 10.0 (0–83.1)	4.7 ± 5.1 (0–51.6)	3.3 ± 4.4 (0–26.9)	0.6 ± 0.9 (0–7.7)
Juvenile female	36	93.6 ± 21.0 (61.5–100)	1.1 ± 2.1 (0–10)	0	4.1 ± 7.0 (0–71.4)	1.2 ± 2.3 (0–8.2)	0	0
Adult female	26	73.4 ± 12.3 (28.8–100)	0.7 ± 4.8 (0–7.1)	5.1 ± 14.4 (0–70)	13.7 ± 20.4 (0–48.9)	4.3 ± 4.1 (0–16.1)	2.4 ± 3.9 (0–13.3)	0.4 ± 1.0 (0–3.9)
Juvenile male	12	93.6 ± 34.8 (0–100)	3.5 ± 2.8 (0–10)	0	2.5 ± 13.8 0–18.0	0.4 ± 1.7 (0–3.2)	0	0
Adult male	58	73.3 ± 10.2 (20–100)	0.7 ± 1.7 (0–3.7)	1.3 ± 4.3 (0–30)	14.7 ± 16.2 (0–83.1)	5.1 ± 9.4 (0–51.6)	4.1 ± 5.8 (0–26.9)	0.8 ± 1.6 (0–7.7)

On the basis of identification of bat flies occurring on *Rousettus madagascariensis* in the same cave [22, 23], it is presumed that the vast majority of bat flies captured and consumed by the host were nycteribiids, specifically *Eucampsipoda madagascariensis*. Further, no evidence was found of invertebrates flying-off during a bat grooming bout, which in this case would be the streblid *Megastrebla wenzeli*; however, it is uncertain if such a detail would have been observable based on the resolution of the video recordings.

The most common behavioral activity of the sampled bats was rest, which represented more than 70% of the time spent for diurnal within the day-roost site, followed by auto-grooming at 15%. No juvenile was observed to engage in a fight or in any form of ectoparasite consumption. For sampled adults, the mean proportion of time allocated to consuming ectoparasites *via* auto-grooming or allo-grooming was 3.3 ± 4.4% (*n* = 84) and 0.6 ± 0.9% (*n* = 84), respectively (Table 2). The mean proportion of time allotted to consuming ectoparasites *via* auto-grooming for adults females and males was 2.4 ± 3.9% (*n* = 26) and 4.1 ± 5.8% (*n* = 58), respectively, and in the case of the consumption *via* allo-grooming 0.4 ± 1.0% (*n* = 26) and 0.8 ± 1.6% (*n* = 58), respectively.

### Correlation between bat grooming behavior and ectoparasites consumption

The mean proportion of time spent by *Rousettus madagascariensis* auto-grooming and consuming ectoparasites were significantly correlated for adults (Spearman’s rank correlation: *r*_*s*_ = 0.552, *P* < 0.001, two-tailed, *n* = 84), as well as the mean proportion of time allocated to allo-grooming and consuming ectoparasites (*r*_*s*_ = 0.353, *P* = 0.004, two-tailed, *n* = 84). The correlation between the mean proportion of time allotted to auto-grooming and consuming ectoparasites in both adult females (*r*_*s*_ = 0.715, *P* < 0.001, two-tailed, *n* = 26) and adult males (*r*_*s*_ = 0.802, *P* < 0.001, two-tailed, *n* = 58) were significant. It was the same case for the relation between the mean proportion of time allocated to allo-grooming and consuming ectoparasites in both adult females (*r*_*s*_ = 0.401, *P* = 0.042, two-tailed, *n* = 26) and adult males (*r*_*s*_ = 0.447, *P* < 0.001, two-tailed, *n* = 58).

### Comparison of proportion of time allocated to grooming and consuming ectoparasites between the age and sex classes

**Fig. 1:**
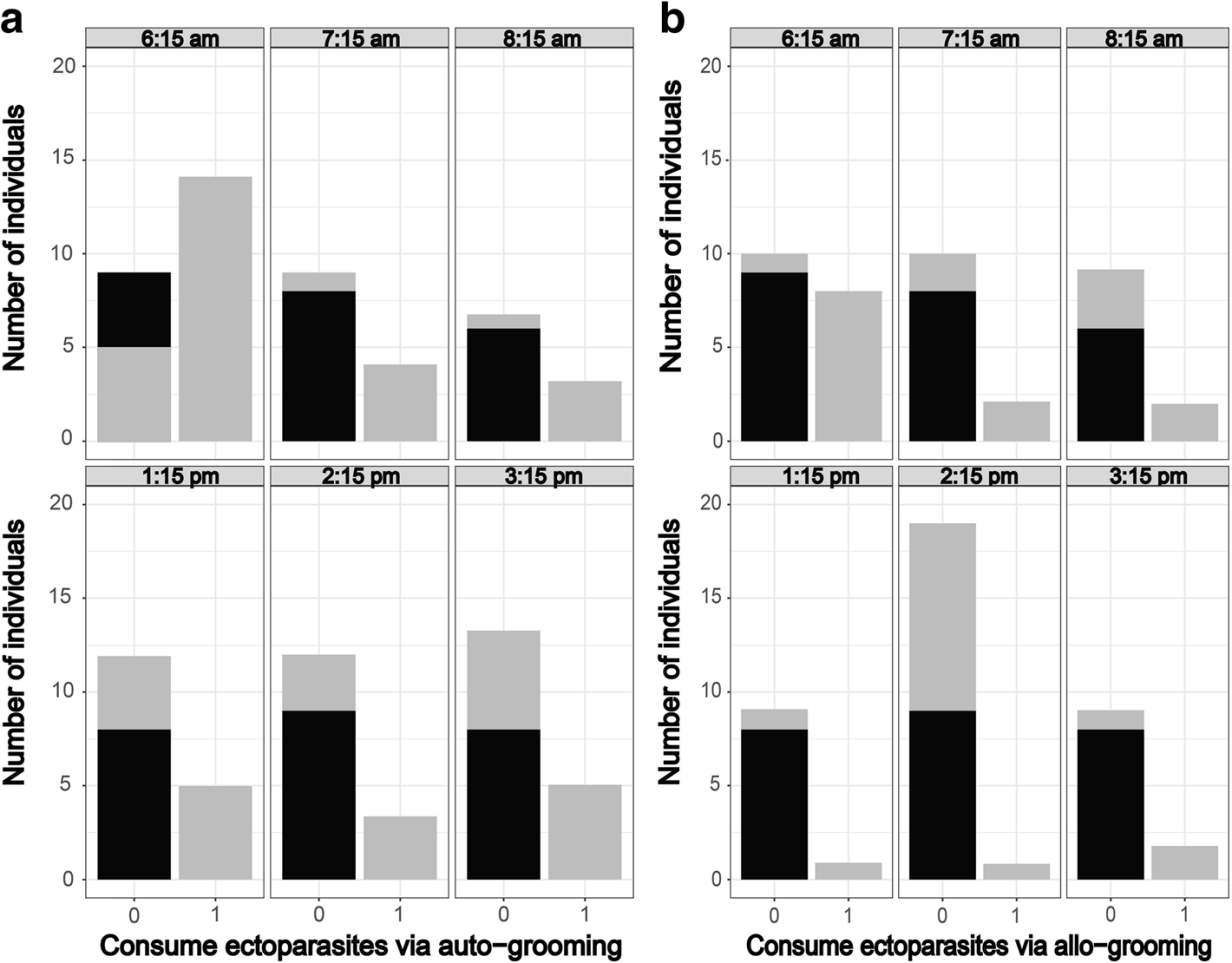
Number of adult and juvenile *Rousettus madagascariensis* consuming ectoparasites *via* auto-grooming (**a**) and allo-grooming (**b**) based on the different daily recording sessions. The X-axis is scored in the following manner: 0 = no ectoparasite was consumed and 1 = at least a single ectoparasite was consumed. Gray coloration is for adults and black for juveniles

**Fig. 2:**
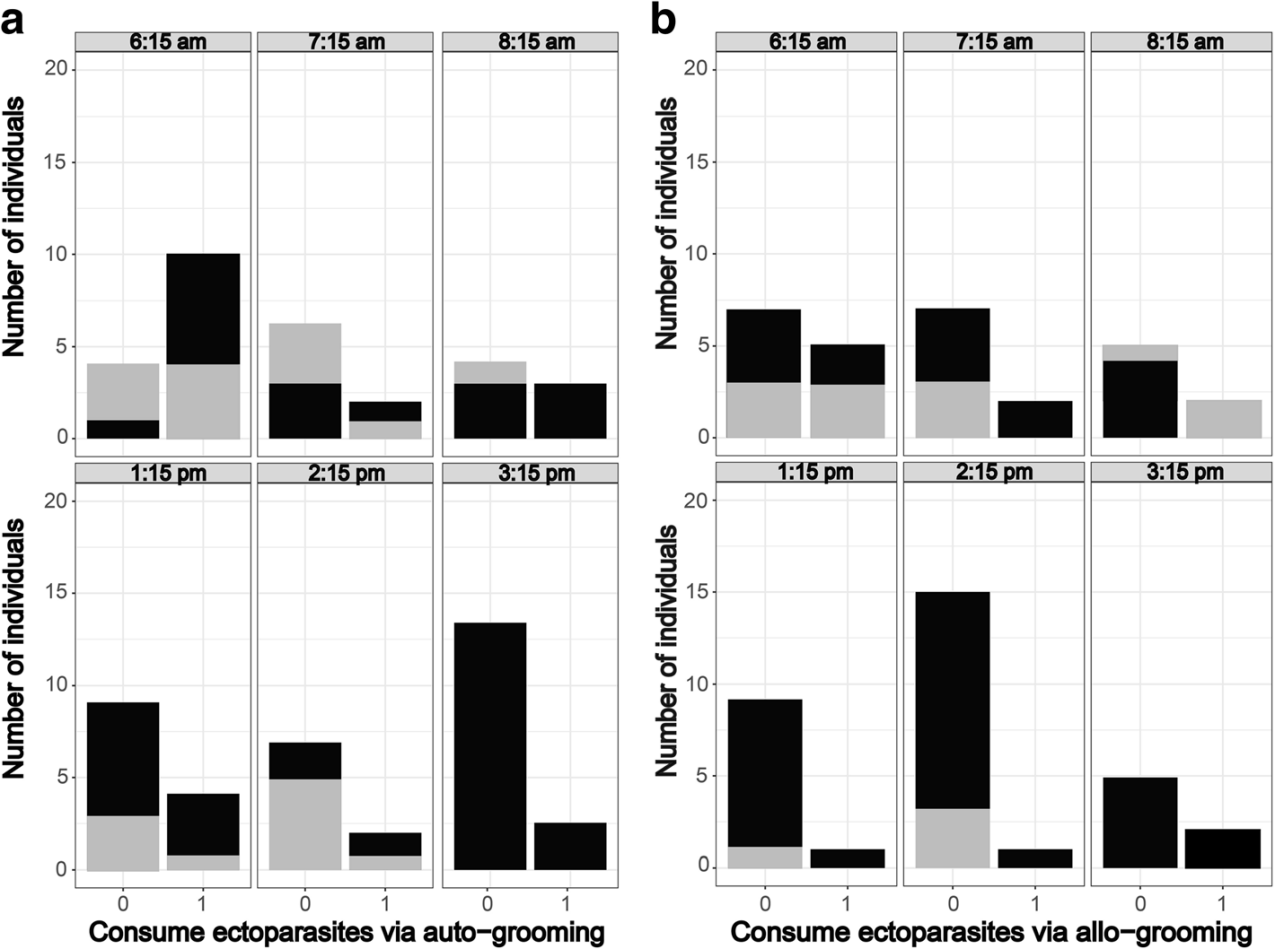
Number of adult female and male *Rousettus madagascariensis* consuming ectoparasites *via* auto-grooming (**a**) and allo-grooming (**b**) based on the different daily recording sessions. The X-axis is scored in the following manner: 0 = no ectoparasite was consumed and 1 = at least a single ectoparasite was consumed. Gray coloration is for females and black for males

The mean proportion of time allocated to auto-grooming in adults was higher than in juveniles (Wilcoxon rank sum test: *W =* 2420, *P* < 0.001, *n* = 132), as well as the time allocated to allo-grooming (Wilcoxon rank sum test: *W =* 2541, *P* < 0.001, *n* = 132). Only adults consumed ectoparasites (Table 2, Fig. 1). There was no significant difference in periods spent in auto-grooming between adult females and males (Wilcoxon rank sum test: *W =* 736, *P* = 0.291, *n* = 84), as well as the amount of time allo-grooming (Wilcoxon rank sum test: *W =* 726, *P* = 0.119, *n* = 84). The difference in the proportion of time apportioned to the consumption of ectoparasites *via* auto-grooming between adult females and males was not significant (Wilcoxon rank sum test: *W =* 591, *P* = 0.101, *n* = 84; Fig. 2a), as was the case *via* allo-grooming (Wilcoxon rank sum test: *W =* 660, *P* = 0.226, *n* = 84; Fig. 2b).

### Differences in ectoparasite consumption in the day roost site

As mentioned in the methods section, daily data recording sessions were divided into six different 10 min periods. The consumption of ectoparasites by adult *Rousettus madagascariensis via* auto-grooming showed no significant differences across the six recording sessions (Kruskall-Wallis test: *χ*^2^_(5)_ = 8.5, *P* = 0.134, *n* = 84). In comparison, the amount of time allocated to ingesting ectoparasites *via* allo-grooming during the 18:15 h session was significantly greater than the other five sessions [Kruskall-Wallis test: *χ*^2^_(5)_ = 13.2, *P* = 0.022, *n* = 84, Dunn *post-hoc* test: 18:15 *vs* 7:15 h (*Z* = 2.4, *P =* 0.019, *n* = 84), 6:15 *vs* 8:15 h (*Z* = 2.9, *P =* 0.003, *n* = 84), 6:15 *vs* 13:15 h (*Z* = 2.8, *P =* 0.004, *n* = 84), 6:15 *vs* 14:15 h (*Z* = 2.4, *P =* 0.019, *n* = 84), 6:15 *vs* 15:15 pm (*Z* = 1.9, *P =* 0.040, *n* = 84)].

We make the assumption that measures of individual bat ectoparasite consumption after returning to the cave (approximately 5:00 h) until the start of the 7:15 h session, are represented from data gathered during the 6:15 h session; this period is referred to herein as roosting period 1. The data obtained for ectoparasite consumption from the other five sessions (7:15 h, 8:15 h, 13:15 h, 14:15 h, and 15:15 h) is assumed to average rates during the balance of the period in the roost and is referred to as roosting period 2. The difference in the time allocated to consuming ectoparasites *via* auto-grooming between roosting period 1 and period 2 was significantly different (Wilcoxon rank sum test: *W* = 799, *P* = 0.008, *n* = 84), as was also found for the consumption of ectoparasites *via* allo-grooming (Wilcoxon rank sum test: *W* = 780, *P* = 0.002, *n* = 84). Estimates of ectoparasites consumption per day was calculated with the average ingestion rates for the two roosting periods.

### Estimation of ectoparasite consumption by *Rousettus*

The number of ectoparasites consumed *via* auto-grooming and allo-grooming was assumed to be constant during roosting period 1 (from 5:00 to 7:14 h) and during the roosting period 2 (7:15 to 18:00 h). During the nine days of behavioral recording, the extrapolated mean total number of ectoparasites that an adult consumed during the roosting period 1 was 14 ± 23 ectoparasites (range 2–67) or about on average 6.2 ectoparasites per hour, as compared to the roosting period 2, which was 23 ± 7 ectoparasites (range 10–30) or about on average 2.1 ectoparasites per hour. The estimated mean number of ectoparasites consumed on a daily basis (about 13 hours) by an individual adult *R*. *madagascariensis* while in the roost site was 37 ± 16 ectoparasites (range 2–67) or 2.8 ectoparasites per hour.

The study period was during the dry season and the number of *R*. *madagascariensis* occupying the day-roost site in September 2016, the same seasonal period, using a capture-mark-recapture technique was estimated as 1908 individuals (95% CI: 675–3517 individuals) [15], and 18% were juveniles [16]. With juveniles removed from the calculations and based on a population of 1565 adults within the day-roost site, using the 37 ectoparasites ingested per day by *R*. *madagascariensis*, the mean number of ectoparasites consumed was 57,905 ± 25,040 ectoparasites (range 18,780–104,855). However, given different aspects of the reproductive strategies of bat flies [28], combined with a prevalence of 97.1% and the mean intensity of 6.6 bat flies per adult host parasitized [24], our inference of daily consumption is certainly overestimated as the estimated bat fly population in the cave is estimated at 10,025 ectoparasites associated with the *R. madagascariensis* colony.

## Discussion

### Diurnal activities of *Rousettus madagascariensis*

Among the different behavioral activities, adults spent more than 70% of their time at rest, followed by approximately 14% in auto-grooming, and 4% in allo-grooming (Table 2). These proportions are similar to those for other non-Malagasy species of bats [20, 29, 30].

### Proportion of time spent grooming and consuming ectoparasites

In the present study, the correlation between the time spent by male and female adults grooming and consuming ectoparasites was significant. Bat flies seem to prefer parasitizing adults, as compared to juveniles, which for *Rousettus madagascariensis* might in part be associated with fur density providing greater protection from host grooming [12, 22]; however, given that juveniles were not observed feeding on ectoparasites, this supposition is called into question. Most filmed individuals were clustered in close groups and often in direct body contact, which would allow direct dispersal of bat flies between them. Although adult males have heavier streblid and nycteribiid parasite loads than adult females throughout the year [22], the amount of time in the day roost associated with the removal of ectoparasites is not significantly different between the sex classes.

In a sort of axiom manner, it would be assumed that ectoparasite consumption by a host animal is a manner to reduce individual ectoparasite loads, although there is an energetic cost to grooming [28, 29]. Another advantage of this behavior would be nutritional, in that about 37 ectoparasites being consumed by individual adult *Rousettus* per day would be a measurable energetic supplement, particularly gravid female bat flies, supplementing protein in a frugivorous species. In general, fruits are rich in carbohydrates and generally low in fats and proteins [31]. This is an interesting aspect to be examined in future research, including nutritional analyses of adult females (with and without pupae) and adult males, to evaluate the potential contribution of ectoparasite consumption in the dietary needs of a frugivorous bat species.

### The periods and rates of daily ectoparasite consumption

Previous studies on non-Malagasy bats [20, 21, 32] showed that bat-grooming activities are more common in the period immediately after bats return to their day-roost sites, as well as before their dusk exit. The filming sessions in the context of the current study do not allow late afternoon activities to be addressed as the last session was at 15:15 h. However, our data indicates a higher rate of grooming and ectoparasite removal in the early morning period.

Previous research on *Rousettus madagascariensis* at the same study site found seasonal differences in the average number of parasites per host, with higher infestation rates during the dry season (June and October) [24], which coincides with the data reported herein. Further, in this bat species, ectoparasite loads are correlated with host sex and age, with adult males having the highest rates [23]. Hence, the data presented herein presumably represent the period with the highest levels of bat fly parasitism rates in *R*. *madagascariensis*.

As mentioned above, the daily estimated consumption by *Rousettus* in the Grotte des Chauves-souris day roost colony of close to 58,000 ectoparasites is simply overestimated. This is presumably related to our extrapolations of consumption rates during periods video recordings were not made and certain cases of recorded mastication that were not associated with the ingestion of bat flies. However, in any case, this study provides new insight into host-ectoparasite interactions and population dynamics of bat flies. These high levels of ectoparasite ingestion underscores new questions if bat fly consumption by the bat host is associated with regulation of their population size associated with body hygiene, a defense mechanism against parasites and/or as a dietary supplement; further, there is the potential role of this behavior in the transmission of zoonotic pathogens to the host. Bacteria of the genus *Bartonella* have been identified in *Eidolon dupreanum*, another endemic Malagasy Pteropodidae and cave-dwelling fruit bat, and from its hematophagous nycteribiid *Cyclopodia dubia* [4]. We suspect that with testing, *R*. *madagascariensis* bat flies will also be found to be positive for *Bartonella.* Further, previous research has shown that bats are more vulnerable to bacterial infections [33], as compared to viral infections, and bacterial pathogens have been isolated from bat flies [4].

## Conclusions

Only adult *Rousettus madagascariensis* were found to consume ectoparasites *via* both auto-grooming and allo-grooming and a significant positive correlation was found between the proportion of time allocated to grooming and ectoparasite consumption in both sexes of adult bats. The considerable estimate of bat fly consumption by their hosts provides a new dimension to population dynamics and reproductive strategies in bat flies. The results presented here will need to be further tested, for example, with a video camcorder apparatus with high resolution to better document physical consumption of ectoparasites and extended across the complete period bats are occupying their day roost. While to our knowledge, rates of bat ingestion of their dipteran ectoparasites has not been previously estimated, the results presented herein lead to a series of questions ranging from the nutritional advantages to the host bat in ectoparasite consumption to possible mechanisms for the transmission of zoonotic diseases *via* an oral route between ectoparasite-host, as compared to blood meals by these hematophagous ectoparasites. These are aspects need to be addressed by future research.
